# Assessment of the End Point Adjudication Process on the Results of the Platelet-Oriented Inhibition in New TIA and Minor Ischemic Stroke (POINT) Trial: A Secondary Analysis

**DOI:** 10.1001/jamanetworkopen.2019.10769

**Published:** 2019-09-06

**Authors:** Mary Farrant, J. Donald Easton, Eric E. Adelman, Brett L. Cucchiara, William G. Barsan, Holly J. Tillman, Jordan J. Elm, Anthony S. Kim, Anne S. Lindblad, Yuko Y. Palesch, Wenle Zhao, Keith Pauls, Kyle B. Walsh, Joan Martí-Fàbregas, Richard A. Bernstein, S. Claiborne Johnston

**Affiliations:** 1 University of California, San Francisco; 2 Department of Neurology, University of Wisconsin School of Medicine and Public Health, Madison; 3 University of Pennsylvania, Philadelphia; 4 Department of Emergency Medicine, University of Michigan, Ann Arbor; 5 Data Coordination Unit, Department of Public Health Sciences, Medical University of South Carolina, Charleston; 6 The Emmes Corporation, Rockville, Maryland; 7 Department of Emergency Medicine, University of Cincinnati, Cincinnati, Ohio; 8 Department of Neurology, Hospital de la Santa Creu i Sant Pau, Barcelona, Spain; 9 Department of Neurology, Feinberg School of Medicine, Northwestern University, Chicago, Illinois; 10 Dean’s Office, Dell Medical School, The University of Texas at Austin

## Abstract

**Importance:**

Debate continues about the value of event adjudication in clinical trials and whether independent centralized assessments improve reliability and validity of study results in masked randomized trials compared with local, investigator-assessed end points.

**Objective:**

To assess the results of the adjudicated end point process in the Platelet-Oriented Inhibition in New TIA and Minor Ischemic Stroke (POINT) trial by comparing end points assessed by local site investigators with centrally adjudicated end points.

**Design, Setting, and Participants:**

This is an ad hoc secondary analysis of a randomized, double-blind clinical trial comparing safety and effectiveness of clopidogrel bisulphate plus aspirin vs placebo plus aspirin. Patients received either 600 mg of clopidogrel bisulphate on day 1, then 75 mg per day through day 90 plus 50 to 325 mg of aspirin per day, or the same range of dosages of placebo plus aspirin. Investigators reported all potential end points; independent masked adjudicators were randomly assigned to review using definitions specified in the study protocol. This was a multicenter study; 269 international sites in 10 countries enrolled from May 28, 2010, to December 19, 2017. The study enrolled 4881 patients 18 years or older with transient ischemic attack or minor acute ischemic stroke within 12 hours of symptom onset and followed for 90 days from randomization; last follow-up was completed in March 2018.

**Main Outcomes and Measures:**

Independent adjudicators external to the study and masked to study treatment assignment adjudicated 467 primary and secondary effectiveness outcomes and major and minor bleeding events, including the primary composite end point, which was the risk of a composite of major ischemic events at 90 days, defined as ischemic stroke, myocardial infarction, or death from an ischemic vascular event. The primary safety end point was major hemorrhage. All components of the primary and safety outcomes were adjudicated.

**Results:**

In this secondary analysis of an international randomized clinical trial, a total of 269 sites worldwide randomized 4881 patients (median age, 65.0 years; interquartile range, 55-74 years); 55.0% were male. The primary results have been published previously. The hazard ratios for clopidogrel plus aspirin vs placebo plus aspirin for the primary composite end point were 0.75 (95% CI, 0.59-0.95) for adjudicator-assessed events and 0.76 (95% CI, 0.60-0.95) for investigator-assessed events. Agreement between adjudicator and investigator assessments was 90.7%. The hazard ratios for clopidogrel plus aspirin vs placebo plus aspirin for the primary safety end point were 2.32 (95% CI, 1.10-4.87) for adjudicator-assessed events and 2.58 (95% CI, 1.19-5.58) for investigator-assessed events, with an agreement rate of 77.5%.

**Conclusions and Relevance:**

Independent end point adjudication did not substantially alter estimates of the primary treatment effectiveness in the POINT trial.

**Trial Registration:**

ClinicalTrials.gov identifier: NCT00991029

## Key Points

**Question:**

Is there an advantage to centrally adjudicating clinical trial end points compared with relying on investigator-assessed end points?

**Findings:**

In this secondary analysis of an international randomized clinical trial of 4881 patients who received clopidogrel bisulphate plus aspirin vs placebo plus aspirin, independent end point adjudication did not substantially change estimates of the primary treatment associations compared with investigator-assessed end points.

**Meaning:**

Independent end point adjudication may have no clinically meaningful improvement on estimates of treatment associations in studies of transient ischemic attack and minor stroke when masking is well controlled and training is provided.

## Teaser

This secondary analysis of an international randomized clinical trial investigates the results of the adjudicated end point process in the Platelet-Oriented Inhibition in New TIA and Minor Ischemic Stroke (POINT) trial by comparing end points assessed by local site investigators with centrally adjudicated end points.

## Introduction

Centralized adjudication may be helpful in reducing variability in safety and effectiveness outcome assessments in studies with complex or subjective end points, high enrollment targets, long duration, or global and cultural differences across sites.^1,2,3,4,5^ In studies where intervention masking at a site level is difficult or uncertain, adjudication can reduce bias associated with perceived knowledge of the treatment assignment.^4,6,7^

Despite thorough prespecified event definitions and established policies, assessments of end points may differ from investigator to investigator. Much attention is given to the accurate classification of these events because large-scale, multicenter studies present particular challenges to the consistent diagnosis of outcome events, and this may alter trial power and treatment effect size.^2,3,4,5^

Although there are no specific requirements or definitive recommendations for ascertaining end points in clinical trials, adjudication finds a basis in the US Food and Drug Administration (FDA) guidance for industry. Regulatory authorities, such as the FDA and the European Medicine Agency, place significant focus on clinical trial processes that ensure consistent, standardized, objective, and unbiased reporting of safety and effectiveness results.^8,9,10,11^ The coordination of outcome adjudication procedures in many multicenter clinical trials remains a manual process, with poor efficiency, high cost, and high risk of delay.^12^ Efforts to automate the adjudication process and minimize end point misclassification in large-scale, multicenter randomized clinical trials (RCTs) have included independent adjudicators masked to study treatments to standardize assessment of outcomes and reduce risk of ascertainment bias of study end points. However, the use of adjudication increases the complexity and cost of a trial, and it is unclear whether the added burden is justified, particularly in double-blind trials.

Adjudication of study end points by independent masked adjudicators provided centralized, standardized, and unbiased assessments in the Platelet-Oriented Inhibition in New TIA and Minor Ischemic Stroke (POINT) trial, a randomized, double-blind clinical trial spanning multiple geographic regions and clinical practice settings. The central aim of this ad hoc analysis was to assess the association of central adjudication by quantifying the proportion of site-reported outcome events also classified as the same event by study adjudicators.

## Methods

### Study Design

The POINT trial was approved by institutional review boards and ethics committees according to local and national regulatory requirements; all patients provided written informed consent. The study design and results have been published previously.^13,14^ This study followed the Consolidated Standards of Reporting Trials (CONSORT) reporting guideline. The trial was stopped early on recommendation of the data and safety monitoring board.

In this multicenter study, 4881 patients at 269 participating international sites in 10 countries who were 18 years or older with either high-risk transient ischemic attack (defined as an ABCD^2^ score of ≥4) or minor acute ischemic stroke (defined as a National Institutes of Health Stroke Scale score ≤3) were randomized within 12 hours of symptom onset. These individuals were enrolled from May 28, 2010, to December 19, 2017, and composed the intent-to-treat (ITT) analysis population. Last follow-up was completed in March 2018.

In a masked fashion, patients were assigned 1:1 to 90 days of clopidogrel bisulfate (600-mg loading dose on day 1, followed by 75 mg per day for days 2-90) or matching placebo. The protocol specified maintenance treatment with open-label aspirin, 50 to 325 mg per day, with actual aspirin dosage at the discretion of the site investigator. The primary composite end point for the trial was the risk of a composite of new major ischemic events at 90 days, including ischemic stroke, myocardial infarction (MI), or death from an ischemic vascular event. The primary safety end point was major hemorrhage within 90 days.

### Event Adjudication

An independent adjudication committee composed of neurologists, cardiologists, and internists led by a committee chair adjudicated primary and secondary effectiveness outcomes and all major and minor bleeding events using supporting documents, translated as necessary. To avoid bias, adjudicators were masked to the arm of the study to which each patient had been randomized. Adjudicators received training on the adjudication process, including outcome event definitions found in eAppendix 1 in the Supplement.

Major hemorrhage other than intracranial hemorrhage was further defined as bleeding that resulted in symptomatic intracranial hemorrhage, intraocular bleeding causing loss of vision, need for transfusion of 2 or more units of red blood cells or equivalent amount of whole blood, need for hospitalization or prolongation of an existing hospitalization, or death. Bleeding events related to surgical procedures were also included.^14^

The Statistics and Data Management Center for the POINT trial, located at the Medical University of South Carolina, developed a web-based clinical trial management system (CTMS) for the study. It included a 6-step outcome adjudication module (Figure), which automated the coordination of adjudication activities by controlling workflows, in real time, based on data collected in the CTMS.^12^

**Figure.**
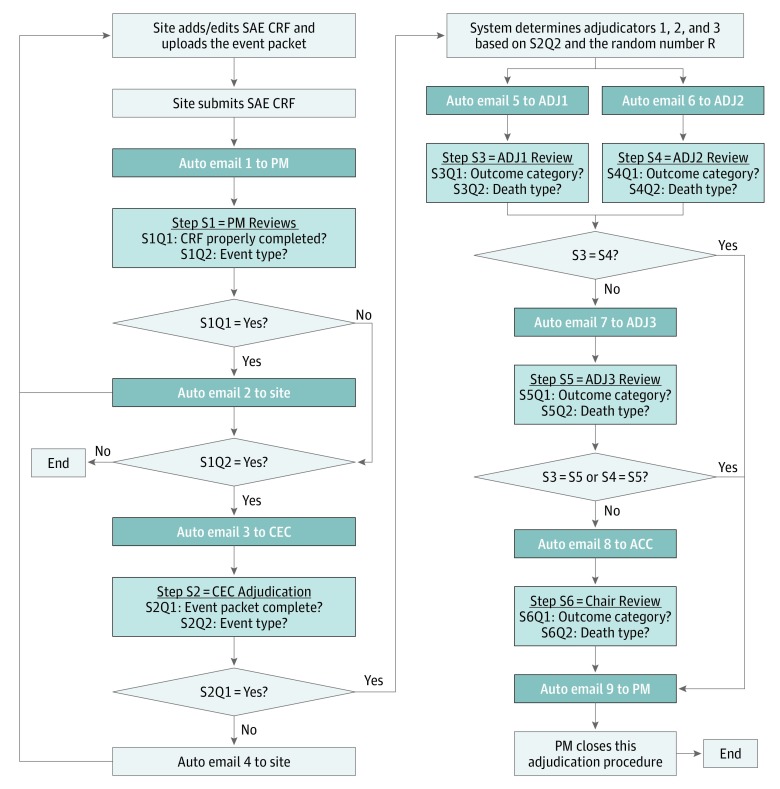
Six-Step Outcome Adjudication Module The web-based clinical trial management system for the Platelet-Oriented Inhibition in New TIA and Minor Ischemic Stroke (POINT) trial automates the coordination of adjudication activities by controlling workflows, in real time, based on data collected. ACC indicates adjudication committee chair; ADJ, adjudicator; CEC, clinician event monitor; CRF, case report form; PM, project manager; Q, question; S, step; and SAE, serious adverse event.

Site investigators reported all suspected outcome events using prespecified standardized definitions described in the protocol and listed on the case report form (eAppendix 2 in the Supplement) and prepared and uploaded an event packet for each suspected end point, with translations provided as needed. The packet consisted of a standardized checklist (eAppendix 3 in the Supplement) and redacted copies of discharge summaries, consultation notes, head imaging reports, and laboratory values for both the index and outcome events, as well as a concise clinical narrative summary prepared by the site investigator.

When a clinical outcome event case report form was submitted by the site and the event packet was considered complete, the system started an independent dual adjudication. Two adjudicators were randomly selected based on the outcome event type (neurologic, cardiac, or systemic) to independently classify the event (and death type if applicable) and record their findings online (eAppendix 4 in the Supplement).

Adjudicators either confirmed or refuted the initial diagnosis reported by site investigators using standardized definitions and the protocol. If discrepancies occurred between the results of the 2 adjudicators, a third adjudicator was notified to independently adjudicate the outcome event. If the third adjudicator disagreed with both previous adjudicators, the adjudication committee chair conducted the final adjudication and entered the results in the CTMS.

The study protocol was updated in 2011 (version 3.0) with a definition of ischemic vascular death as death due to ischemic stroke, MI, sudden cardiac death, arrhythmia, pulmonary embolism, bowel or limb infarction, or any death not readily attributable to a nonischemic cause. However, the corresponding clinical outcome reporting form was not updated to include ischemic vascular death as a choice, despite its being a component of the primary composite end point, so sites could not specify it as an outcome event. During the analysis phase of the study, a post hoc interpretation of the variable was contrived whereby an outcome was considered an investigator-assessed ischemic vascular death if the outcome was fatal and the investigator selected ischemic stroke or MI as the outcome on the reporting form.

The results of the adjudicated end point process were evaluated by comparing the primary findings of adjudicator-assessed end points with investigator-assessed end points; the rate of agreement between the findings was calculated for each outcome. All analyses were completed according to the ITT principle.

### Statistical Analysis

The POINT trial adjudicators adjudicated 467 primary and secondary effectiveness outcomes and major and minor bleeding events, including the primary composite end point and the primary safety end point. Time from randomization to the first end point of interest was calculated for both treatment groups using the log-rank test; the hazard ratios (HRs) and 95% CIs were estimated using a Cox proportional hazards regression model. For each outcome, plus the individual components of the primary composite end point, agreement between adjudicator-assessed and investigator-assessed end points was calculated as the percentage of end points equivalently classified by sites and adjudicators.

SAS software (version 9.4; SAS Institute Inc) was used to perform all statistical analyses. Statistical significance was set at 2-sided *P* < .05.

## Results

In this secondary analysis of an international RCT, a total of 269 sites randomized 4881 patients (median age, 65.0 years; interquartile range, 55-74 years); 55.0% were male. The primary results have been published previously.^13,14^

### Primary Composite End Point

There were 467 events adjudicated in the POINT trial. Of these, the primary composite end point of major ischemic event occurred in 281 patients, including 121 of 2432 patients (5.0%) receiving clopidogrel plus aspirin and 160 of 2449 patients (6.5%) receiving placebo plus aspirin. The treatment benefit observed with clopidogrel plus aspirin was similar using adjudicator-assessed or investigator-assessed events. The HRs for clopidogrel plus aspirin vs placebo plus aspirin for the primary composite end point were 0.75 (95% CI, 0.59-0.95; *P* = .02) for adjudicator-assessed events and 0.76 (95% CI, 0.60-0.95; *P* = .02) for investigator-assessed events (Table 1).

**Table 1.** Primary Composite End Point and Primary Safety End Point at 90 Days After Randomization for All Adjudicated Events

Outcome Type	Assessment Type	Total Events, No.	Total Patients With Event, No.	Patients With Event, No. (Event Rate, %)		Hazard Ratio (95% CI)	*P* Value by Log-Rank Test	Agreement, %
				Clopidogrel Plus Aspirin (n = 2432)	Placebo Plus Aspirin (n = 2449)			
Primary composite end point (ischemic stroke, myocardial infarction, and ischemic vascular death)	Adjudicated	298	281	121 (5.0)	160 (6.5)	0.75 (0.59-0.95)	.02	90.7
	Investigator	320	299	129 (5.3)	170 (6.9)	0.76 (0.60-0.95)	.02	
Ischemic stroke	Adjudicated	278	267	112 (4.6)	155 (6.3)	0.72 (0.56-0.92)	.008	91.4
	Investigator	302	287	122 (5.0)	165 (6.7)	0.74 (0.58-0.93)	.01	
Myocardial infarction	Adjudicated	17	17	10 (0.4)	7 (0.3)	1.44 (0.55-3.78)	.46	94.4
	Investigator	18	18	10 (0.4)	8 (0.3)	1.26 (0.50-3.19)	.63	
Ischemic vascular death	Adjudicated	10	10	6 (0.2)	4 (0.2)	1.51 (0.43-5.35)	.52	58.3
	Investigator	9	9	5 (0.2)	4 (0.2)	1.26 (0.34-4.69)	.73	
Primary safety end point (major hemorrhage)	Adjudicated	36	33	23 (0.9)	10 (0.4)	2.32 (1.10-4.87)	.02	77.5
	Investigator	35	32	23 (0.9)	9 (0.4)	2.58 (1.19-5.58)	.01	
Primary composite end point (ischemic stroke and myocardial infarction only)	Adjudicated	295	278	119 (4.9)	159 (6.5)	0.75 (0.59-0.95)	.02	91.6
	Investigator	320	299	129 (5.3)	170 (6.9)	0.76 (0.60-0.95)	.02	

The HRs for the association of randomized treatment with ischemic stroke were 0.72 (95% CI, 0.56-0.92; *P* = .008) for adjudicator-assessed strokes and 0.74 (95% CI, 0.58-0.93; *P* = .01) for investigator-assessed strokes (Table 1). The HRs for MI were 1.44 (95% CI, 0.55-3.78; *P* = .46) for adjudicator-assessed events and 1.26 (95% CI, 0.50-3.19; *P* = .63) for investigator-assessed events. The HRs were 1.51 (95% CI, 0.43 to 5.35; *P* = .52) for adjudicator-assessed ischemic vascular deaths and 1.26 (95% CI, 0.34-4.69; *P* = .73) for investigator-assessed ischemic vascular deaths.

Overall, comparisons of the primary composite end point determination between adjudicators and site investigators showed an agreement rate of 90.7% (Table 1). Agreement between the adjudicator-assessed and investigator-assessed events was also calculated for the individual components of the primary composite end point, with concordance high for both ischemic stroke (91.4%) and MI (94.4%). Agreement was lower for ischemic vascular death, at 58.3%. Given the small number of events adjudicated in this category, the HRs for the primary composite end point were recalculated after the deaths were removed; there was a slight shift in concordance from 90.7% to 91.6% after their removal (Table 1). Ischemic vascular death showed little association with the treatment effectiveness compared with the original primary composite end point.

### Primary Safety End Point

A total of 33 patients in the POINT ITT population had at least 1 major hemorrhage (Table 2), 23 (0.9%) in the clopidogrel plus aspirin group and 10 (0.4%) in the placebo plus aspirin group. Three patients experienced 2 major hemorrhages (upper gastrointestinal in 2 patients and lower gastrointestinal in 1 patient). As summarized in Table 1, the HRs were 2.32 (95% CI, 1.10-4.87; *P* = .02) for adjudicator-assessed major hemorrhage and 2.58 (95% CI, 1.19-5.58; *P* = .01) for investigator-assessed major hemorrhage. Agreement between adjudicator-assessed and investigator-assessed safety end points was 77.5% (Table 1).

**Table 2.** Major and Minor Hemorrhages for the POINT Trial Intent-to-Treat Study Population

Outcome	Patients With Event, No. (%)		Hazard Ratio (95% CI)	*P* Value
	Clopidogrel Plus Aspirin (n = 2432)	Placebo Plus Aspirin (n = 2449)		
Fatal major hemorrhage^a^	3 (0.1)	2 (0.1)	NA	NA
Major hemorrhage	23 (0.9)	10 (0.4)	2.32 (1.10-4.87)	.02
Intracranial hemorrhage	6 (0.2)	3 (0.1)	NA	NA
Hemorrhagic stroke	5 (0.2)	3 (0.1)	NA	NA
Symptomatic intracerebral hemorrhage	2 (0.1)	2 (0.1)	NA	NA
Symptomatic hemorrhagic transformation of cerebral infarcts	2 (0.1)	1 (0.0)	NA	NA
Other symptomatic intracranial hemorrhage (subarachnoid)	2 (0.1)	0	NA	NA
Other than intracranial hemorrhage^b^	17 (0.7)	7 (0.3)	NA	NA
Upper GI hemorrhage	4 (0.2)	4 (0.2)	NA	NA
Lower GI hemorrhage	7 (0.3)	1 (0.0)	NA	NA
Hematuria	1 (0.0)	1 (0.0)	NA	NA
Other^c^	5 (0.2)	1 (0.0)	NA	NA
Minor hemorrhage	40 (1.6)	13 (0.5)	3.12 (1.67-5.83)	<.001

Abbreviations: GI, gastrointestinal; NA, not applicable; POINT, Platelet-Oriented Inhibition in New TIA and Minor Ischemic Stroke.

^a^ The 5 fatal major hemorrhages were 2 symptomatic intracerebral hemorrhages, 2 symptomatic hemorrhagic transformations, and 1 groin hemorrhage with cardiac arrest.

^b^ Three of these patients had 2 hemorrhagic events each.

^c^ Includes 1 uterine fibroid, 1 vitreous hemorrhage, 1 implantation of a loop recorder, 1 gallbladder hematoma, and 1 groin hemorrhage with cardiac arrest. One patient had traumatic arm hemorrhage due to a fall adjudicated as a major hemorrhage; according to protocol, this did not qualify as a major hemorrhage because it was due to trauma.

## Discussion

The findings from the POINT trial contribute to the continuing discussion about the value of end point adjudication in large-scale double-blind, placebo-controlled, randomized trials. Outcome event adjudication can be helpful in harmonizing and standardizing outcome assessment in studies like the POINT trial with complex end points, high enrollment targets, long duration, and differences across sites.^1,2,3,4,5^ While it has been suggested that having an event adjudication process in place often encourages greater confidence in trial results from regulatory authorities and clinicians, several recent meta-analyses of RCTs suggest that the use of adjudicators might be most important when site investigators are not masked to treatment assignment and the risk of misclassification is high.^14^

A Cochrane review^3^ published in 2016 compared the results of a systematic review and meta-analysis of treatment association estimates for 47 randomized trials to assess whether there was a difference between the findings obtained by adjudicators and site investigators. The review article found that, on average, treatment association estimates did not differ (combined ratio of the odds ratios, 1.00; 95% CI, 0.97-1.04) and raised concerns about whether adjudication was being used appropriately across RCTs.^3^

A smaller pooled analysis of 10 cardiovascular outcomes trials found that the end point adjudication process had no improvement on the association estimates (ratio of the odds ratios, 1.00; 95% CI, 0.97-1.02) and no difference in the results between masked and unmasked trials.^6^ The findings from a review article of 6 RCTs concluded that adjudication did not improve the ability to determine treatment associations.^7^ Similar observations of consistent treatment associations have been described for a variety of single trials, including ADVANCE,^15^ ENOS,^16^ PREVENU,^17^ and STABILITY.^18^ These findings are compatible with the results from earlier trials, such as PROGRESS^19^ and ACTION,^20^ and raise the question of the utility of adjudication, particularly because the process places considerable resource burdens on trials as clinical research costs continue to escalate.^5,21^

The POINT trial used an independent masked adjudication committee in a randomized, double-blind, placebo-controlled trial design in an attempt to improve accuracy of estimates of treatment association. A rigorous and careful site and investigator selection process for participation in the study was implemented. Investigators used the prespecified definitions (ie, “hard” outcomes) provided in the study protocol, training materials, and case report forms, drawing also from clinical practice, to correctly diagnose the majority of events.

Low rates of concordance between adjudicators and site investigators can raise concerns about the quality and validity of treatment association estimates. Large multicenter trials like the POINT trial can be at risk of outcome misclassification caused by differential application of end point definitions by site investigators. Site investigators may diagnose greater disease severity than adjudicators based on their knowledge of patient symptoms not available to the adjudicators.

In the present analysis of adjudication, agreement between local investigators and adjudicators was highest for the components of MI (94.4%) and ischemic stroke (91.4%). The low concordance rate of 58.3% for ischemic vascular death was not unexpected due to the lack of reporting using a strict definition for this component and because of the small number of events. As stated earlier, despite its being included in the primary composite end point, ischemic vascular death was not a choice on the clinical outcome reporting form; therefore, sites could not specify it as an outcome. Given the small number of events adjudicated in this category, the HRs for the primary composite end point were recalculated after the deaths were removed; there was a slight shift in concordance from 90.7% to 91.6% after their removal (Table 1). Ischemic vascular death showed little influence on the treatment association compared with the original primary composite end point. Where the concordance was low in the POINT trial, it indicated an opportunity to improve end point definitions for future trials.

As noted, subjective outcomes and masked interventions are among reasons offered for continuing to adjudicate outcome events. There may be other reasons, some of them less quantifiable, to include adjudication in the study design, such as providing reassurance to users of trial data and conformity with regulatory requirements. The value of end point adjudication in large-scale randomized trials will continue to be debated as long as regulatory agencies recommend its inclusion in the study design. Alternative, more cost-effective means of end point adjudication that do not compromise the validity and reliability of the results warrant further research.

### Limitations

This analysis encountered some of the more common limitations found in clinical research study design and processes, including early stopping of the trial by the data and safety monitoring board, a lower-than-expected overall event rate yielding fewer primary outcome events than planned, and issues with certain outcome definitions. The adjudication workflows did not include a quality control process to resubmit a fraction of the already adjudicated events to verify the consistency of assessments by individual adjudicators and measure variability throughout the study period. The generic centralized outcome adjudication module did not allow adjudicators to communicate directly with site investigators for additional information or data clarification.

We did not determine a quantitative threshold for the minimum acceptable agreement rate between the treatment association estimates of adjudicators and site investigators to measure what the process could reasonably be expected to contribute to trial conclusions. Little has been written about an “optimal” rate of concordance, although some references recommend 80% as the most common minimum acceptable estimate of ratings between assessors, indicating substantial agreement.^22,23^

We did not develop a formal estimate of the total cost of adjudication. However, with the development of the generic centralized outcome adjudication module, dedicated database, data collection, document translation, and adjudicator payments, it is likely that the overall process represented one of the major trial expenses. Novel tools and emerging technologies capable of driving research efficiencies, such as automated predictive algorithms and machine learning methods, suggest a new era in clinical trial processes.

## Conclusions

Independent masked end point adjudication did not substantially alter estimates of the primary treatment associations compared with investigator-assessed end points in the POINT trial. The findings from the POINT trial, other multicenter large-scale double-blind RCTs, and recent meta-analyses demonstrate continuing uncertainty about the value of end point adjudication and the justification for the increased resource burden of the process.

The reassurance that the adjudication process brings to study findings may be important. However, adjudication may have no meaningful improvement on estimates of treatment association when site personnel are carefully trained, standard reproducible outcome definitions are used, and masking is maintained.

## Notes

Supplement. **eAppendix 1.** Outcome Event Definitions **eAppendix 2.** Clinical Outcome Event Case Report Form **eAppendix 3.** Outcome-Specific Checklist for Preparing Event Packets **eAppendix 4.** Adjudicator Screens/Case Report Form
