# Supplementary material for: Assessment of the End Point Adjudication Process on the Results of the Platelet-Oriented Inhibition in New TIA and Minor Ischemic Stroke (POINT) Trial: A Secondary Analysis
Source: JAMA Netw Open. 2019 Sep 6;2(9):e1910769. doi: 10.1001/jamanetworkopen.2019.10769 (PMC6735409; doi:10.1001/jamanetworkopen.2019.10769)
Supplement: Supplement. — eAppendix 1. Outcome Event Definitions eAppendix 2. Clinical Outcome Event Case Report Form eAppendix 3. Outcome-Specific Checklist for Preparing Event Packets eAppendix 4. Adjudicator Screens/Case Report Form [file jamanetwopen-2-e1910769-s001.pdf]

Supplementary Online Content

Farrant M, Easton JD, Adelman EE, et al. Assessment of the end point adjudication process on the results of the Platelet-Oriented Inhibition in New TIA and Minor Ischemic Stroke (POINT) trial: a secondary analysis. *JAMA Netw Open*. 2019;2(9):e1910769. doi:10.1001/jamanetworkopen.2019.10769

eAppendix 1. Outcome Event Definitions

eAppendix 2. Clinical Outcome Event Case Report Form

eAppendix 3. Outcome-Specific Checklist for Preparing Event Packets

eAppendix 4. Adjudicator Screens/Case Report Form

This supplementary material has been provided by the authors to give readers additional information about their work.

© 2019 Farrant M et al. *JAMA Network Open*.

eAppendix 1. Outcome Event Definitions

Ischemic stroke: An acute focal infarction of the brain or retina (and does not include anterior ischemic optic neuropathy (AION)).

Criteria:

(1) Rapid onset of a new focal neurological deficit with clinical or imaging evidence of infarction and not attributable to a non-ischemic etiology (not associated with brain infection, trauma, tumor, seizure, severe metabolic disease, or degenerative neurological disease); or

(2) Rapid worsening of an existing focal neurological deficit that is judged by the Investigator to be attributable to a new infarction. Criteria for symptoms attributable to new infarction may include symptoms that persist and are judged by the investigator to be attributable to new infarction, imaging evidence of infarction or no evidence of a non-ischemic etiology.

TIA: A neurological deficit of sudden onset, resolving completely, attributed to focal brain or retinal ischemia without evidence of associated acute focal infarction of the brain.

Criteria: Rapid onset of a focal neurological deficit that is without evidence of acute focal infarction of the brain, and is not attributable to a non-ischemic etiology (brain infection, trauma, tumor, seizure, severe metabolic disease, or degenerative neurological disease).

Symptomatic hemorrhagic transformation of an ischemic stroke: Any extravascular blood within an area of known acute/subacute infarction which is judged to be non-traumatic, and responsible for neurologic symptoms. To be considered symptomatic, the hemorrhagic transformation must be judged to be partially responsible for the subject's clinical neurologic presentation (i.e., the area of Infarction is not adequate to explain the neurologic deficit, or a secondary neurologic deterioration occurred corresponding to the timing of hemorrhagic transformation).

Criteria (must meet both of the following):

a. Imaging evidence (by CT or MRI) of extravascular blood within the area of infarction.

b. Symptoms judged to be related to the hemorrhagic transformation. Scenarios which may be judged as symptomatic: (i) If blood is already present on imaging at presentation, symptoms are out of proportion to what would be expected for the size and location of the infarct at presentation; (ii) Clinical deterioration, defined by an increase of 4 points or more in the score on the NIHSS or leading to death, occurring after the initial ischemic event, and identified as the result of the hemorrhagic transformation; or (iii) Mass effect secondary to the hemorrhagic transformation causing symptoms.

© 2019 Farrant M et al. *JAMA Network Open*.

Asymptomatic hemorrhagic transformation of an ischemic stroke: Any extravascular blood within an area of known acute/subacute infarct, judged to be non-traumatic, without any related neurologic symptoms.

Criteria (must meet both of the following):

a. Imaging evidence (by CT or MRI) of extravascular blood within the area of infarct.

b. No symptoms related to the hemorrhagic transformation or clinical deterioration with less than a 4-point increase in score on the NIHSS judged to be related to the hemorrhagic transformation.

Symptomatic intracerebral hemorrhage: Any extravascular blood in the brain parenchyma, judged to be non-traumatic, and not in the area of an acute/subacute ischemic infarct, associated with and identified as the predominant cause of new neurologic symptoms (including headache) or death. In the case of a mixed intracranial hemorrhage [Intracerebral Hemorrhage (ICH), Subarachnoid Hemorrhage (SAH, Subdural Hemorrhage (SDH), and/or Intraventricular Hemorrhage (IVH)], the event should be classified according to the primary site of hemorrhage by the judgment of the clinician. For example, if a patient has a large ICH with a small amount of SAH, and the ICH is felt to be the primary site of bleeding, this should be classified as ICH.

Criteria: Evidence of hemorrhage in the brain parenchyma demonstrated by head imaging, surgery, or autopsy, which is not in the same territory of an underlying acute or subacute ischemic stroke, and is judged to be associated with any new neurologic symptoms (including headache) or leading to death.

Asymptomatic intracerebral hemorrhage: An acute extravasation of blood into the brain parenchyma, judged to be non-traumatic, and not in an area of an acute/subacute ischemic infarct, without associated neurologic symptoms or leading to death. In the case of a mixed intracranial hemorrhage (ICH, SAH, SDH and/or IVH), the event should be classified according to the primary site of hemorrhage by the judgment of the clinician. For example, if a patient has a large ICH with a small amount of SAH, and the ICH is felt to be the primary site of bleeding, this should be classified as ICH.

Criteria: Evidence of hemorrhage in the brain parenchyma demonstrated by head imaging, surgery, or autopsy, which is not in the same territory of an underlying acute or subacute ischemic stroke, and is not judged to be associated with any new neurologic symptoms or leading to death.

© 2019 Farrant M et al. *JAMA Network Open*.

Other symptomatic intracranial hemorrhage: Any extravascular blood within the cranium judged to be non-traumatic, and the predominant cause of the clinical deterioration or that led to death. Other Intracranial Hemorrhage is defined as an acute extravasation of blood into the subarachnoid space, epidural space, subdural space or intraventricular space with associated symptoms (including headache). In the case of a mixed intracranial hemorrhage (ICH, SAH, SDH and/or IVH), the event should be classified according to the primary site of hemorrhage by the judgment of the clinician. For example, if a patient has a large ICH with a small amount of SAH, and the ICH is felt to be the primary site of bleeding, this should be classified as ICH.

> **Criteria:** Evidence of hemorrhage in the subarachnoid space, epidural space, or subdural space demonstrated by head imaging, surgery, or autopsy.

Other asymptomatic intracranial hemorrhage: An acute extravasation of blood into the subarachnoid space, epidural space, subdural space or intraventricular space without associated symptoms, and judged to be non-traumatic. In the case of a mixed intracranial hemorrhage (ICH, SAH, SDH and/or IVH), the event should be classified according to the primary site of hemorrhage by the judgment of the clinician. For example, if a patient has a large ICH with a small amount of SAH, and the ICH is felt to be the primary site of bleeding, this should be classified as ICH.

> **Criteria:** Evidence of hemorrhage in the subarachnoid space, epidural space, or subdural space demonstrated by head imaging, surgery, or autopsy.

Myocardial infarction with coronary revascularization: Evidence of myocardial necrosis in a clinical setting consistent with myocardial ischemia treated with coronary revascularization, such as angioplasty/stenting or coronary artery bypass graft (CABG), within 14 days.

> **Criteria:** The diagnosis of MI will be based on an algorithm developed from the Universal Definition of Myocardial Infarction (Circulation 2007 116:2634-2653) that takes into account 5 categories of clinical information from the acute event: rise and/or fall of cardiac biomarkers, ECG abnormalities, clinical setting, imaging evidence, and pathology.

Myocardial infarction without coronary revascularization: Evidence of myocardial necrosis in a clinical setting consistent with myocardial ischemia not treated with coronary revascularization within 14 days.

> **Criteria:** The diagnosis of MI will be based on an algorithm developed from the Universal Definition of Myocardial Infarction (Circulation 2007 116:2634-2653) that takes into account 5 categories of clinical information from the acute event: rise and/or fall of cardiac biomarkers, ECG abnormalities, clinical setting, imaging evidence, and pathology.

Coronary revascularization without myocardial infarction: A procedure to improve coronary blood flow for documented coronary artery disease, but with no documentation of new post-randomization myocardial infarction. Criteria: Documented coronary angioplasty, stenting, or bypass surgery for demonstrated or presumed coronary artery disease.

© 2019 Farrant M et al. *JAMA Network Open*.

Major hemorrhage other than intracranial hemorrhage (life-threatening or non-life-threatening): A hemorrhagic event, judged to be non-traumatic, that results in intraocular bleeding causing loss of vision, the need for a transfusion of two or more units of red cells or the equivalent amount of whole blood, or the need for hospitalization or prolongation of existing hospitalization. This may include bleeding events related to surgical procedures but not those related to accidental trauma. Life-threatening hemorrhagic events will be defined as those that are fatal or require use of intravenous inotropic medication to maintain blood pressure, interventional treatment (including surgical, endoscopic or endovascular interventions), or transfusion of four or more units of red cells or the equivalent amount of whole blood. Non-life-threatening hemorrhagic events will be defined as those classified as major hemorrhagic events but not as life-threatening.

Minor hemorrhage other than intracranial hemorrhage: All hemorrhagic events leading to interruption or discontinuation of the study drug but not classifiable as major hemorrhagic events. This may include bleeding events related to surgical procedures but not those related to accidental trauma.

Ischemic vascular death: Death due to ischemic stroke, myocardial infarction, sudden cardiac death, arrhythmia, pulmonary embolism, bowel or limb infarction, or any death not readily attributable to a non-ischemic cause.

Hemorrhagic Vascular death: Death due to intracranial or systemic hemorrhage.

Other Serious Adverse Event: Any adverse event, not belonging to the other outcome event categories, that is fatal or life threatening, is permanently or substantially disabling, requires or prolongs hospitalization, results in a congenital anomaly, or requires intervention to prevent permanent impairment or damage.

© 2019 Farrant M et al. *JAMA Network Open*.

eAppendix 2. Clinical Outcome Event Case Report Form

Form 19: SAE/Clinical Outcome Reporting Form (Version 9)

This CRF should only be completed if the subject experiences a Serious Adverse Event (SAE) or Clinical Outcome after the enrolling/index event. This form should be data entered and submitted within 5 days of discovery.

An Outcome Event Visit should be conducted if a subject experiences an ischemic stroke, TIA, or myocardial infarction. Outcome Event Visits can be done via telephone unless the subject experiences an ischemic stroke or TIA, in which case an in-person visit should be conducted.

Worsening of an enrolling stroke is an outcome event.

1	Name of SAE/Clinical outcome: (100 character max)	
2	Date of onset:	______ -__________-_____________(dd-mmm-yyyy)
3	Time of onset: (For clinical outcome events, this is the time the deficit was first recognized.)	______ :_______(24 hour clock, hh:mm)

General Comments:

Name of person who collected these data (not for data entry):

Signature of Reviewing Investigator/Date (not for data entry):

© 2019 Farrant M et al. *JAMA Network Open*.

POINT version 9 13Oct2014

Clinical outcomes / SAEs

If the event listed in Question 1 matches any of these clinical outcomes/ SAEs, mark the appropriate circle.

O **Ischemic stroke:** An acute focal infarction of the brain or retina (and does not include anterior ischemic optic neuropathy (AION)). Criteria: (1) Rapid onset of a new focal neurological deficit with clinical or imaging evidence of infarction and not attributable to a non-ischemic etiology (not associated with brain infection, trauma, tumor, seizure, severe metabolic disease, or degenerative neurological disease); or, (2) Rapid worsening of an existing focal neurological deficit that is judged by the Investigator to be attributable to a new infarction. Criteria for symptoms attributable to new infarction *may* include symptoms that persist and are judged by the investigator to be attributable to new infarction, imaging evidence of infarction or no evidence of a non- ischemic etiology.

O **TIA:** A neurological deficit of sudden onset, resolving completely, attributed to focal brain or retinal ischemia without evidence of associated acute focal infarction of the brain. Criteria: rapid onset of a focal neurological deficit that is without evidence of acute focal infarction of the brain, and is not attributable to a non-ischemic etiology (brain infection, trauma, tumor, seizure, severe metabolic disease, or degenerative neurological disease).

O **Symptomatic hemorrhagic transformation of an ischemic stroke:** Any extravascular blood within an area of known acute/subacute infarction which is judged to be nontraumatic, and responsible for neurologic symptoms. To be considered symptomatic, the hemorrhagic transformation must be judged to be partially responsible for the subject's clinical neurologic presentation (i.e., the area of Infarction is not adequate to explain the neurologic deficit, or a secondary neurologic deterioration occurred corresponding to the timing of hemorrhagic transformation). Criteria (must meet both of the following):

a. Imaging evidence (by CT or MR) of extravascular blood within the area of infarction.
b. Symptoms judged to be related to the hemorrhagic transformation. Scenarios which may be judged as symptomatic: (i) If blood is already present on imaging at presentation, symptoms are out of proportion to what would be expected for the size and location of the infarct at presentation; (ii) Clinical deterioration, defined by an increase of 4 points or more in the score on the NIHSS or leading to death, occurring after the initial ischemic event, and identified as the result of the hemorrhagic transformation; or (iii) Mass effect secondary to the hemorrhagic transformation causing symptoms.

O **Asymptomatic hemorrhagic transformation of an ischemic stroke:** Any extravascular blood within an area of known acute/subacute infarct, judged to be nontraumatic, without any related neurologic symptoms. Criteria (must meet both of the following):

a. Imaging evidence (by CT or MRI) of extravascular blood within the area of infarct.
b. No symptoms related to the hemorrhagic transformation, or clinical deterioration with less than a 4-point increase in score on the NIHSS judged to be related to the hemorrhagic transformation.

O **Symptomatic intracerebral hemorrhage:** Any extravascular blood in the brain parenchyma, judged to be nontraumatic, and not in the area of an acute/subacute ischemic infarct, associated with and identified as the predominant cause of new neurologic symptoms (including headache) or death. In the case of a mixed intracranial hemorrhage [Intracerebral Hemorrhage (ICH), Subarachnoid Hemorrhage (SAH, Subdural Hemorrhage (SDH), and/or Intraventricular Hemorrhage (IVH)], the event should be classified according to the primary site of hemorrhage by the judgment of the clinician. For example, if a patient has a large ICH with a small amount of SAH, and the ICH is felt to be the primary site of bleeding, this should be classified as ICH. Criteria: Evidence of hemorrhage in the brain parenchyma demonstrated by head imaging, surgery, or autopsy, which is not in the same territory of an underlying acute or subacute ischemic stroke, and is judged to be associated with any new neurologic symptoms (including headache) or leading to death.

O **Asymptomatic intracerebral hemorrhage:** An acute extravasation of blood into the brain parenchyma, judged to be nontraumatic, and not in an area of an acute/subacute ischemic infarct, without associated neurologic symptoms or leading to death. In the case of a mixed intracranial hemorrhage (ICH, SAH, SDH and/or IVH), the event should be classified according to the primary site of hemorrhage by the judgment of the clinician. For example, if a patient has a large ICH with a small amount of SAH, and the ICH is felt to be the primary site of bleeding, this should be classified as ICH. Criteria: evidence of hemorrhage in the brain parenchyma demonstrated by head imaging, surgery, or autopsy, which is not in the same territory of an underlying acute or subacute ischemic stroke, and is not judged to be associated with any new neurologic symptoms or leading to death.

O **Other symptomatic intracranial hemorrhage:** Any extravascular blood within the cranium judged to be nontraumatic, and the predominant cause of the clinical deterioration or that led to death. Other Intracranial Hemorrhage is defined as an acute extravasation of blood into the subarachnoid space, epidural space, subdural space or intraventricular space with associated symptoms (including headache). In the case of a mixed intracranial hemorrhage (ICH, SAH, SDH and/or IVH), the event should be classified according to the primary site of hemorrhage by the judgment of the clinician. For example, if a patient has a large ICH with a small amount of SAH, and the ICH is felt to be the primary site of bleeding, this should be classified as ICH. Criteria: Evidence of hemorrhage in the subarachnoid space, epidural space, or subdural space demonstrated by head imaging, surgery, or autopsy.

O **Other asymptomatic intracranial hemorrhage:** An acute extravasation of blood into the subarachnoid space, epidural space, subdural space or intraventricular space without associated symptoms, and judged to be nontraumatic. In the case of a mixed intracranial hemorrhage (ICH, SAH, SDH and/or IVH), the event should be classified according to the primary site of hemorrhage by the judgment of the clinician. For example, if a patient has a large ICH with a small amount of SAH, and the ICH is felt to be the primary site of bleeding, this should be classified as ICH. Criteria: Evidence of hemorrhage in the subarachnoid space, epidural space, or subdural space demonstrated by head imaging, surgery, or autopsy.

O **Myocardial infarction with coronary revascularization:** Evidence of myocardial necrosis in a clinical setting consistent with myocardial ischemia treated with coronary revascularization, such as angioplasty/stenting or coronary artery bypass graft (CABG), within 14 days. Criteria: The diagnosis of MI will be based on an algorithm developed from the Universal Definition of Myocardial Infarction (Circulation 2007 116:2634-2653) that takes into account 5 categories of clinical information from the acute event: rise and/or fall of cardiac biomarkers, ECG abnormalities, clinical setting, imaging evidence, and pathology.

O **Myocardial infarction without coronary revascularization:** Evidence of myocardial necrosis in a clinical setting consistent with myocardial ischemia not treated with coronary revascularization within 14 days. Criteria: The diagnosis of MI will be based on an algorithm developed from the Universal Definition of Myocardial Infarction (Circulation 2007 116:2634-2653) that takes into account 5 categories of clinical information from the acute event: rise and/or fall of cardiac biomarkers, ECG abnormalities, clinical setting, imaging evidence, and pathology.

O **Coronary revascularization without myocardial infarction:** A procedure to improve coronary blood flow for documented coronary artery disease, but with no documentation of new post-randomization myocardial infarction. Criteria: Documented coronary angioplasty, stenting, or bypass surgery for demonstrated or presumed coronary artery disease.

O **Major hemorrhage other than intracranial hemorrhage (life-threatening or non-life-threatening):** A hemorrhagic event, judged to be nontraumatic, that results in intraocular bleeding causing loss of vision, the need for a transfusion of two or more units of red cells or the equivalent amount of whole blood, or the need for hospitalization or prolongation of existing hospitalization. This may include bleeding events related to surgical procedures but not those related to accidental trauma. Life-threatening hemorrhagic events will be defined as those that are fatal or require use of intravenous inotropic medication to maintain blood pressure, interventional treatment (including surgical, endoscopic or endovascular interventions), or transfusion of four or more units of red cells or the equivalent amount of whole blood. Non-life-threatening hemorrhagic events will be defined as those classified as major hemorrhagic events but not as life-threatening.

O **Minor hemorrhage other than intracranial hemorrhage:** All hemorrhagic events leading to interruption or discontinuation of the study drug but not classifiable as major hemorrhagic events. This may include bleeding events related to surgical procedures but not those related to accidental trauma.

O **Other serious adverse event:** Any adverse event, not belonging to the other outcome event categories, that is fatal or life threatening, is permanently or substantially disabling, requires or prolongs hospitalization, results in a congenital anomaly, or requires intervention to prevent permanent impairment or damage.

© 2019 Farrant M et al. *JAMA Network Open*.

15	If 'Symptomatic hemorrhagic transformation of an ischemic stroke' or 'Asymptomatic hemorrhagic transformation of an ischemic stroke', specify:	○ Of Index stroke<br>○ Of Outcome stroke
16	If 'Other symptomatic intracranial hemorrhage' or 'Other asymptomatic intracranial hemorrhage', specify:	○ Subarachnoid hemorrhage<br>○ Subdural hemorrhage<br>○ Intraventricular hemorrhage
17	If 'Myocardial infarction with coronary revascularization' or 'Coronary revascularization without myocardial infarction', specify:	○ Angioplasty/stent<br>○ Coronary Artery Bypass Graft
18	If 'Major Hemorrhage other than Intracranial Hemorrhage', specify:	○ Gastrointestinal<br>○ Genitourinary ○ Other
19	If 'Other Major Hemorrhage other than Intracranial Hemorrhage', specify:	
20	If 'Minor hemorrhage other than intracranial hemorrhage', specify:	○ Gastrointestinal<br>○ Genitourinary ○ Oral<br>○ Cutaneous<br>○ Other
21	If 'Other Minor hemorrhage other than intracranial hemorrhage', specify:	
5	Did the clinical outcome event meet the definition of serious? This should be skipped for non-clinical outcome events (when question 4 = "Other serious adverse event")<br><br>Serious is defined as fatal or life threatening, is permanently or substantially disabling, requires or prolongs hospitalization, results in a congenital anomaly, or requires intervention to prevent permanent impairment or damage.	○ No ○ Yes
6	Severity:<br><br>Severity is used to describe the intensity (severity) of a specific event (as in mild myocardial infarction vs. severe myocardial infarction).	○ Mild<br>○ Moderate<br>○ Severe<br>○ Life threatening / Disabling<br>○ Fatal
General Comments:		
Name of person who collected these data (not for data entry):		
Signature of Reviewing Investigator/Date (not for data entry):		

POINT version 9 13Oct2014

© 2019 Farrant M et al. *JAMA Network Open*.

7	Outcome:	○ Resolved<br>○ Resolved w/ sequellae<br>○ Continuing (Follow up is required)<br>○ Continuing at end of study (No follow up is required)<br>○ Continuing at time of death
8	Date of resolution/death:	______ -__________-_____________(dd-mmm-yyyy)
9	What is the relationship of the SAE to the study drug?<br><br>Q9 should only be answered if Q4 is '**Other serious adverse event'.**	○ Not related<br>The temporal relationship between treatment exposure and the adverse event is unreasonable or incompatible and/or adverse event is clearly due to extraneous causes (e.g., underlying disease, environment)<br>○ Unlikely (must have 2)<br>• Could readily have been produced by the subject's clinical state, or environmental or other interventions.<br>• Does not follow known pattern of response to intervention.<br>• Does not reappear or worsen with reintroduction of intervention.<br>○ Possibly (must have 2)<br>• Has a reasonable temporal relationship to intervention.<br>• Could not readily have been produced by the subject's clinical state or environmental or other interventions.<br>• Follows a known pattern of response to intervention.<br>○ Probably (must have 3)<br>• Has a reasonable temporal relationship to intervention.<br>• Could not readily have been produced by the subject's clinical state or have been due to environmental or other interventions.<br>• Follows a known pattern of response to intervention.<br>• Disappears or decreases with cessation of intervention.<br>○ Definitely (must have all 4)<br>• Has a reasonable temporal relationship to intervention.<br>• Could not readily have been produced by the subject's clinical state or have been due to environmental or other interventions.<br>• Follows a known pattern of response to intervention.<br>• Disappears or decreases with cessation of intervention and recurs with re-exposure.
General Comments:		
Name of person who collected these data (not for data entry):		
Signature of Reviewing Investigator/Date (not for data entry):		

POINT version 9 13Oct2014

© 2019 Farrant M et al. *JAMA Network Open*.

		☐ None	☐ Other medication change
10	What actions were taken for this event? (Check all that apply)	☐ Study drug reduced<br>☐ Study drug held	☐ Procedure/Surgery<br>☐ Hospitalization/Prolonged hosp.
		☐ Study drug discontinued	☐ Unknown
11	Describe the event in detail:<br><br>Include a description of what happened and a summary of all relevant clinical information (medical status prior to the event, signs and/or symptoms, differential diagnosis for the event in question, clinical course, treatment outcome, etc)<br><br>DO NOT identify any study participant, physician, or institution by name.		
12	Relevant tests /laboratory data (both positive and negative), including dates:		
13	Last name of reviewing site investigator		
14	Date of site investigator review	______ -__________- ____________	(dd-mmm-yyyy)

Please note that Event Packets must be uploaded for all Clinical Outcome Events and SAEs.
The Site/Hub PI will work with the Site Manager to prepare Event Packets, including copies of discharge summaries, neurology, cardiology or other consultation notes, head imaging reports, appropriate laboratory values, and a narrative summary, with all unique identifiers removed.

The first page of all event packets should include the event packet checklist, indicating which procedures/tests/notes/etc. are contained in the event packet. In rare cases where no information was collected for the event packet, the event packet checklist must be uploaded indicating that no information is available and the reason why. For more information regarding the Event Packet please refer to the POINT MoP.

General Comments:

Name of person who collected these data (not for data entry):

Signature of Reviewing Investigator/Date (not for data entry):

© 2019 Farrant M et al. *JAMA Network Open*.

eAppendix 3. Outcome-Specific Checklist for Preparing Event Packets

**POINT Clinical Outcome-Specific Checklist for Preparing Event Packets**				
**Please use this form as a face page, and order the Event Packet documents in the order in which they appear below.**				
NOTE: All protected health information (PHI) must be removed from documents (Event Packets must be de-identified).				
**Category:**	**Checklist Item**	**Submitted**	**Not Done**	**Done but Unavailable***
**Basic Packet for ALL Events:**	Clinical Outcome Reporting Form (CRF 19)	☐	☐	☐
	Discharge Summary (Index Event)	☐	☐	☐
	Discharge Summary (Outcome Event)	☐	☐	☐
	All Head Imaging Reports (Index Event)	☐	☐	☐
	All Head Imaging Reports (Outcome Event)	☐	☐	☐
	Consult Notes (neurology, cardiology, etc.)	☐	☐	☐
**Depending on event category, also include the following documents in the packet:**				
**All Deaths**	Autopsy Report	☐	☐	☐
Includes Fatal SAEs	Death Certificate	☐	☐	☐
	Emergency Team/Ambulance Report	☐	☐	☐
	Nursing Home Report	☐	☐	☐
**Ischemic Stroke**	Carotid Imaging Report	☐	☐	☐
With or Without Hemorrhagic Transformation	Operative Report	☐	☐	☐
**TIA***	Carotid Imaging Report	☐	☐	☐
*Not adjudicatable				
**Intracranial Hemorrhage**	Operative Report	☐	☐	☐
(Symptomatic ICH,				
Asymptomatic ICH, Other				
Symp ICranialHem & Other				
Asymp ICranial Hem)				
**Cardiac Outcomes**	All Cardiac Enzyme Reports	☐	☐	☐
(MI with or without	ECG Report(s)	☐	☐	☐
Coronary Revasc)				
**Hemorrhage Other than**	Number of Transfusions	☐	☐	☐
**Intracranial (Major & Minor)**	Operative Report	☐	☐	☐
			*If unavailable, explain	
**Comments:**				

© 2019 Farrant M et al. *JAMA Network Open*.

eAppendix 4. Adjudicator Screens/Case Report Form

Project Manager (PM) Screen: PM reviews packet for completeness; PM can query the site directly through WebDCU.

Review Step	Reviewer	Date	Record Version	Q1	Q2	Comments	Action
PM Completeness Review				Report type: ○ New Event Report ○ Follow-up Report ○ Data Correction	Requires review by CEC: ○ No ○ Yes	*Limit to 1000 char.*	Save Cancel

Clinician Event Monitor (CEM) Screen: CEM reviews event packet for *completeness*, *event seriousness/expectedness/relationship to study drug;* and *type of outcome*; CEM can query the site directly through WebDCU.

Review Step	Reviewer	Date	Record Version	Q1	Q2	Q3	Q4	Q5	Comments	Action
CEC Review				Event Packet Complete: ○ No ○ Yes	Serious: ○ No ○ Yes	Unexpected? ○ No ○ Yes	Relationship to Study Drug: ○ Not related ○ Unlikely ○ Possibly ○ Probably ○ Definitely	Type: ○ Neurological ○ Systemic ○ Cardiac ○ N/A	*Limit to 1000 char.*	Save Cancel

© 2019 Farrant M et al. *JAMA Network Open*.

Adjudicator Screen: Adjudicator reviews packet for *adjudicated outcome* and *type of death (if fatal)*; adjudicators can ask the CEM to query sites on their behalf.

Review Step	Reviewer	Date	Record Version	Q1	Q2	Comments	Action
Adjudicator Review				Adjudicated Outcome =	If Q6 (Severity) is fatal, what type of death?<br>○ Ischemic Vascular Death Neurological<br>○ Hemorrhagic Vascular Death<br>○ Non-vascular death<br>○ Not Fatal	*Limit to 1000 char.*	Save<br>Cancel

Options for Q1 Adjudicated Outcome:

Please Select

- Please Select
- Asymptomatic hemorrhagic transformation of an ischemic stroke
- Asymptomatic intracerebral hemorrhage
- Coronary Revascularization without Myocardial Infarction
- Ischemic stroke
- Major Hemorrhage other than Intracranial Hemorrhage
- Minor hemorrhage other than intracranial hemorrhage
- Myocardial infarction with Coronary Revascularization
- Myocardial Infarction without Coronary Revascularization
- No Event
- Other Asymptomatic intracranial hemorrhage
- Other Serious Adverse Event
- Other Symptomatic intracranial hemorrhage
- Symptomatic hemorrhagic transformation of an ischemic stroke
- Symptomatic intracerebral hemorrhage
- TIA

© 2019 Farrant M et al. *JAMA Network Open*.
